# Iron oxide nanoparticles carried by probiotics for iron absorption: a systematic review

**DOI:** 10.1186/s12951-023-01880-9

**Published:** 2023-04-10

**Authors:** Călina Ciont, Amalia Mesaroș, Oana Lelia Pop, Dan Cristian Vodnar

**Affiliations:** 1 Department of Food Science, University of Agricultural Sciences and Veterinary Medicine, 400372 Cluj-Napoca, Romania; 2 Molecular Nutrition and Proteomics Laboratory, Institute of Life Sciences, University of Agricultural Sciences and Veterinary Medicine, 400372 Cluj-Napoca, Romania; 3 Institute of Life Sciences, University of Agricultural Sciences and Veterinary Medicine, Calea Mănăştur 3–5, 400372 Cluj-Napoca, Romania; 4 Physics and Chemistry Department, C4S Centre, Technical University of Cluj-Napoca, 28 Memorandumului Street, 400114 Cluj-Napoca, Romania; 5 Molecular Nutrition and Proteomics Laboratory, Institute of Life Sciences, University of Agricultural Sciences and Veterinary Medicine Cluj-Napoca, Cluj-Napoca, Romania

**Keywords:** Iron oxide nanoparticles, Cytotoxicity, Probiotics, Absorption, Drug delivery, Anemia

## Abstract

**Background:**

One-third of the world's population has anemia, contributing to higher morbidity and death and impaired neurological development. Conventional anemia treatment raises concerns about iron bioavailability and gastrointestinal (GI) adverse effects. This research aims to establish how iron oxide nanoparticles (IONPs) interact with probiotic cells and how they affect iron absorption, bioavailability, and microbiota variation.

**Methods:**

Pointing to the study of the literature and developing a review and critical synthesis, a robust search methodology was utilized by the authors. The literature search was performed in the PubMed, Scopus, and Web of Science databases. Information was collected between January 2017 and June 2022 using the PRISMA (Preferred Reporting Items for Systematic Review and Meta-Analysis) protocols for systematic reviews and meta-analyses. We identified 122 compatible research articles.

**Results:**

The research profile of the selected scientific articles revealed the efficacy of IONPs treatment carried by probiotics versus conventional treatment. Therefore, the authors employed content assessment on four topics to synthesize previous studies. The key subjects of the reviewed reports are the characteristics of the IONPs synthesis method, the evaluation of cell absorption and cytotoxicity of IONPs, and the transport of IONPs with probiotics in treating anemia.

**Conclusions:**

To ensure a sufficient iron level in the enterocyte, probiotics with the capacity to attach to the gut wall transport IONPs into the enterocyte, where the maghemite nanoparticles are released.

**Graphical Abstract:**

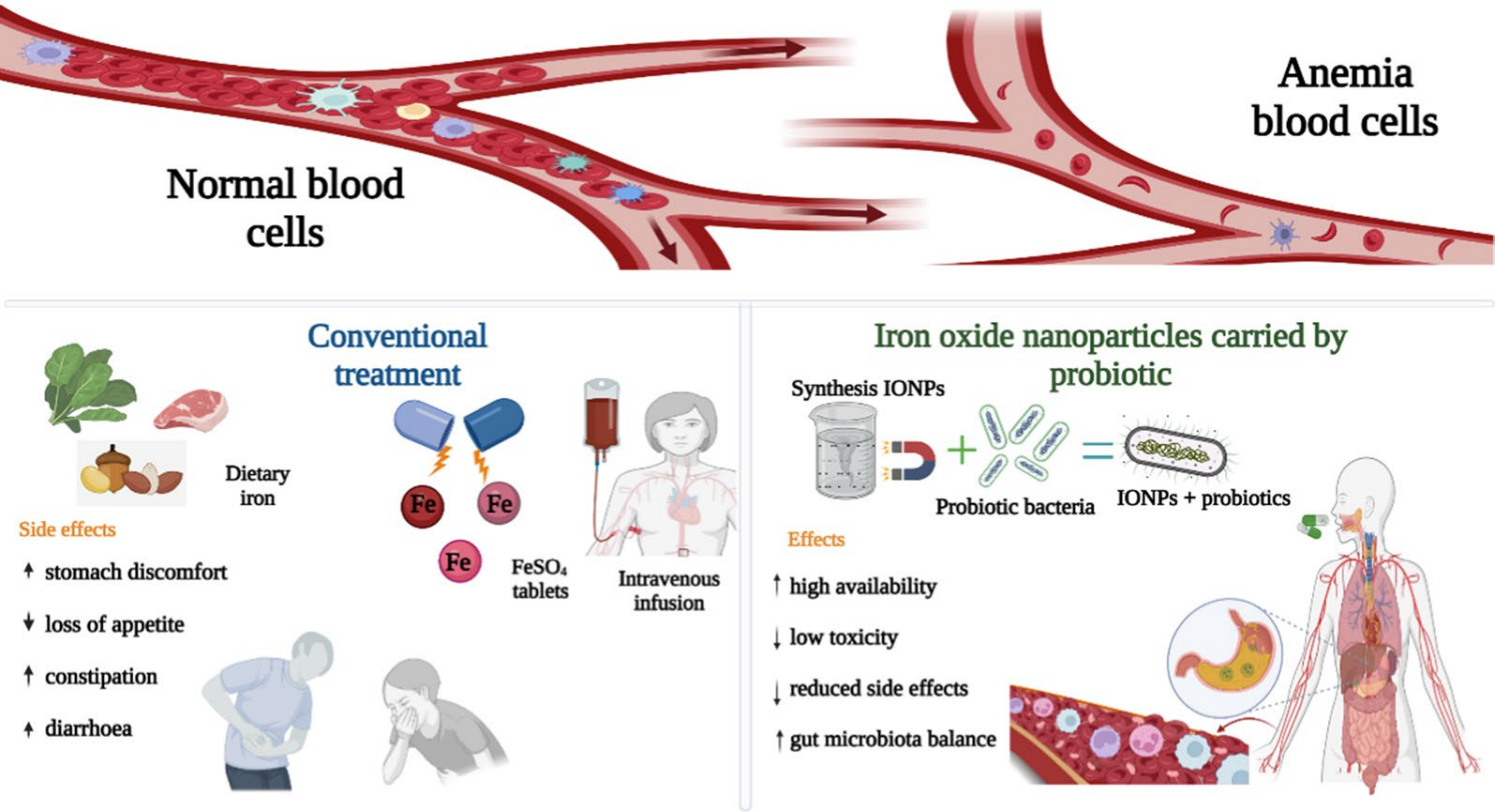

## Background

Iron deficiency anemia (IDA), which is characterized by a hemoglobin level of < 10.0 g/dL, is associated with learning issues, weakness, and an increased risk of comorbidities, such as contracting infections and mortality [1, 2]. The World Health Organization estimates that approximately 24.8% (1.62 billion people) of the world’s population has anemia [3], with children, adolescents, and young/pregnant women most prone to the condition [4, 5]. IDA has several etiologies: (i) inadequate iron consumption, (ii) insufficient pathological assimilation, and (iii) chronic blood loss [6]. Genetic iron overload, characterized by iron accumulation and induced oxidative damage, can lead to life-threatening conditions [7, 8]. Foods fortified with iron can help decrease IDA incidence [9, 10]. However, the most bioavailable water-soluble medicines in this setting, particularly ferrous sulfate (FeSO_4_), sodium iron ethylene diamine [3, 4], and ferrous bis-glycinate chelate [11], introduce unpleasant sensory modifications to the food and impact the gut microbiota [12, 13]. Most of the ingested iron, especially from oral supplements, remains unabsorbed in the intestinal lumen after entering the colon [12, 14], where it can produce free radicals [4]. Intensification of the pathogenic *Enterobacteriaceae* and additional intestinal inflammatory markers are suggested to reduce the proportion of beneficial bacteria, including *Bifidobacterium* and *Lactobacillus* species, in infants receiving iron supplementation [15].

Recently, newly generated iron oxide nanoparticles (IONPs) have been recommended as innovative supplements compared with conventional IDA treatments because of their low reactivity, high bioavailability [12], physical stability, biocompatibility, and ecologically friendly nature [16, 17]. In general, IONPs of < 10 nm exhibit superparamagnetic behavior [18]. Conversely, iron oxide (predominantly magnetite) is hydrophobic and rapidly oxidized in air [19]. External coatings stabilize IONPs in biological environments while limiting magnetism loss [20, 21]. The biodistribution, pharmacokinetics, and suitability of the particles for various biomedical applications are affected by their composition, size, shape, and interference chemistry; these properties are mainly determined by the method of synthesis applied [22, 23].

To better understand the effects of IONPs, cellular endpoints, including apoptosis, mitochondrial viability, and oxidative stress rates, have been studied [24–26]. IONPs have been shown to lead to local and systemic inflammation, oxidative damage, and genotoxicity [26–28]. IONPs induce lower oxidative stress than FeSO_4_ because of their lower absorption [29], which might be explained by the high exposed amounts of Fe^2+^/Fe^3+^ on the prominent surface of IONPs [26, 29, 30]. “Iron overloading” in the intestinal tract may have a significant impact on the species and abundance of the microbial components of the digestive tract [12, 31].

Probiotic bacteria are essential for maintaining a normal microbiota and can generate a variety of antioxidants and immunological stimulants [32]. The European Food Safety Authority recently reported that probiotics improved iron absorption [33]. *L. fermentum* and *B. breve* have been discovered as platforms with a dense distribution of small IONPs on their exterior surfaces [34]. Treatment with these bacteria together with iron supplements can improve the bioavailability of the nanoparticles [35] and lead to survival from stomach diseases [8, 36].

For many years, side effects to IDA treatment have been discussed without focusing on the solutions of these effects [37–39]. This review aims to understand the interaction between IONPs and probiotic cells, the impact of these interactions on iron absorption, bioavailability, microbiota balance, and their dynamic side effects, and study the emerging nanobiotechnology solutions using new and innovative approaches for IDA prevention and treatment.

First, we designed a congruent study-extraction approach as a theoretical framework, comprising database identification, keyword selection, actual searching, and shortlisting of the relevant studies. Second, we developed a research assessment process to provide comprehensive data on the publication frequency and sources. Third, we applied a manual qualitative approach to distinguish the topics of these publications, and consequently identified four themes were identified regarding IONPs: synthesis, metabolism and cellular absorption, cytotoxicity, and the carrying by probiotic bacteria. Then, we identified research gaps and suggested future directions. Finally, we explored the study’s theoretical and practical consequences and limitations when applying the findings.

Therefore, to support further study of this topic, scientific literature has been assessed and the accumulated content synthesized so that future studies can be developed and ultimately improve the quality of studies conducted in this field. We aimed to pursue the following research objectives (O): O1, examine the research profile of studies; O2, determine, comprehend, and appraise the focus areas of the current literature on the interaction among the probiotics of IONPs; O3, critically evaluate emerging approaches, purposely emphasize incongruity in the present scientific literature, and propose probable research questions; and O4, design a framework that researchers can use to comprehend the outline of IONPs probiotic systems.

## Results

### Study selection and characteristics

**Fig. 1:**
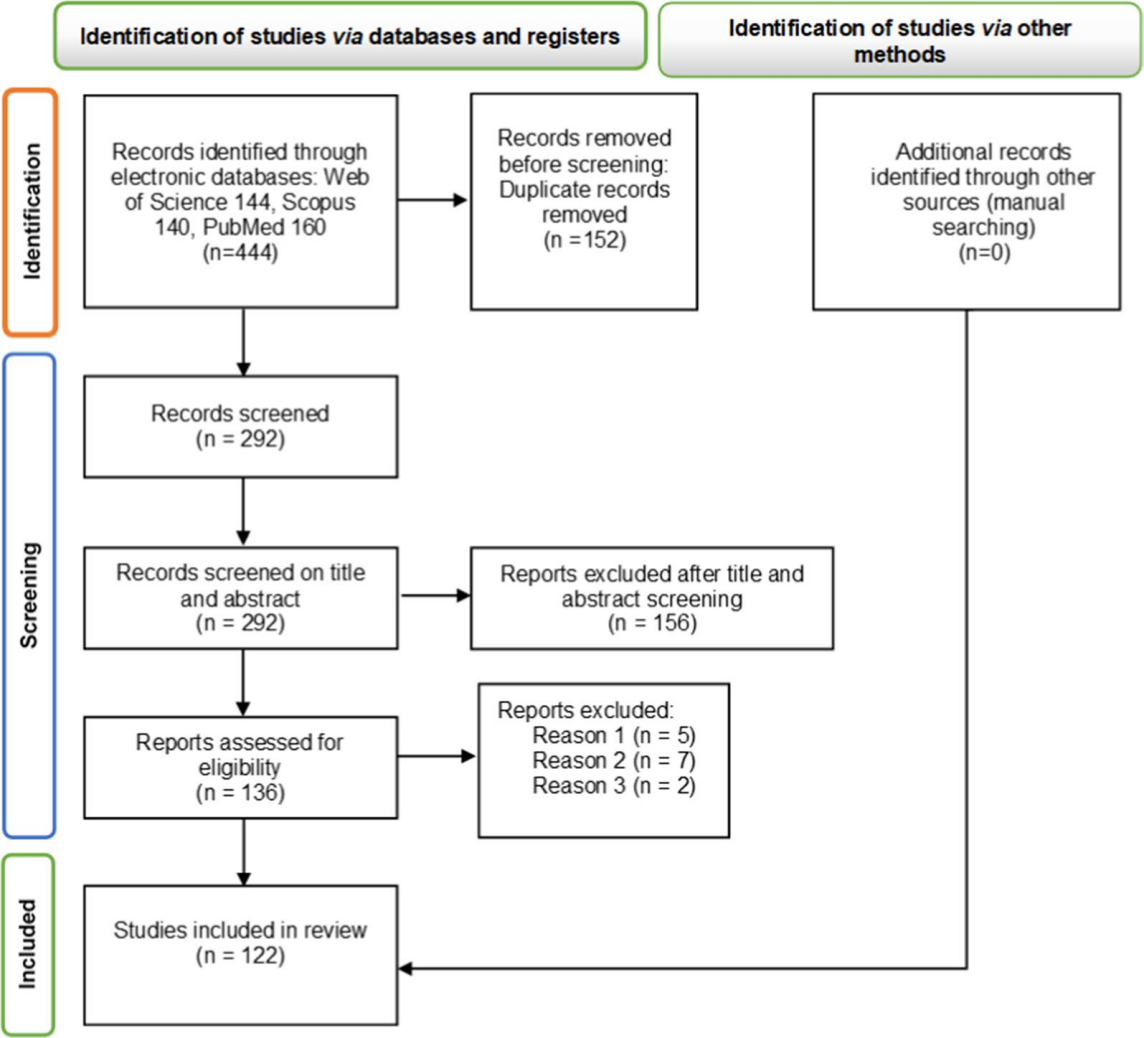
PRISMA flow diagram detailing the study screening and selection procedures

From the preliminary database search, 144, 140, and 160 articles were retrieved from the Web of Science, Scopus, and PubMed, respectively. Of these, 152 were excluded as they were duplicate entries and 156 were excluded after examination of the title and abstract; 136 publications were selected for a comprehensive full-text analysis. After the full manuscript was read and in accordance with the established inclusion and exclusion criteria, 122 manuscripts pertaining to the relationship between probiotics and IONPs were selected for detailed assessment. The Preferred Reporting Items for Systematic reviews and Meta-Analyses (PRISMA) screening process is depicted in Fig. 1.

**Fig. 2:**
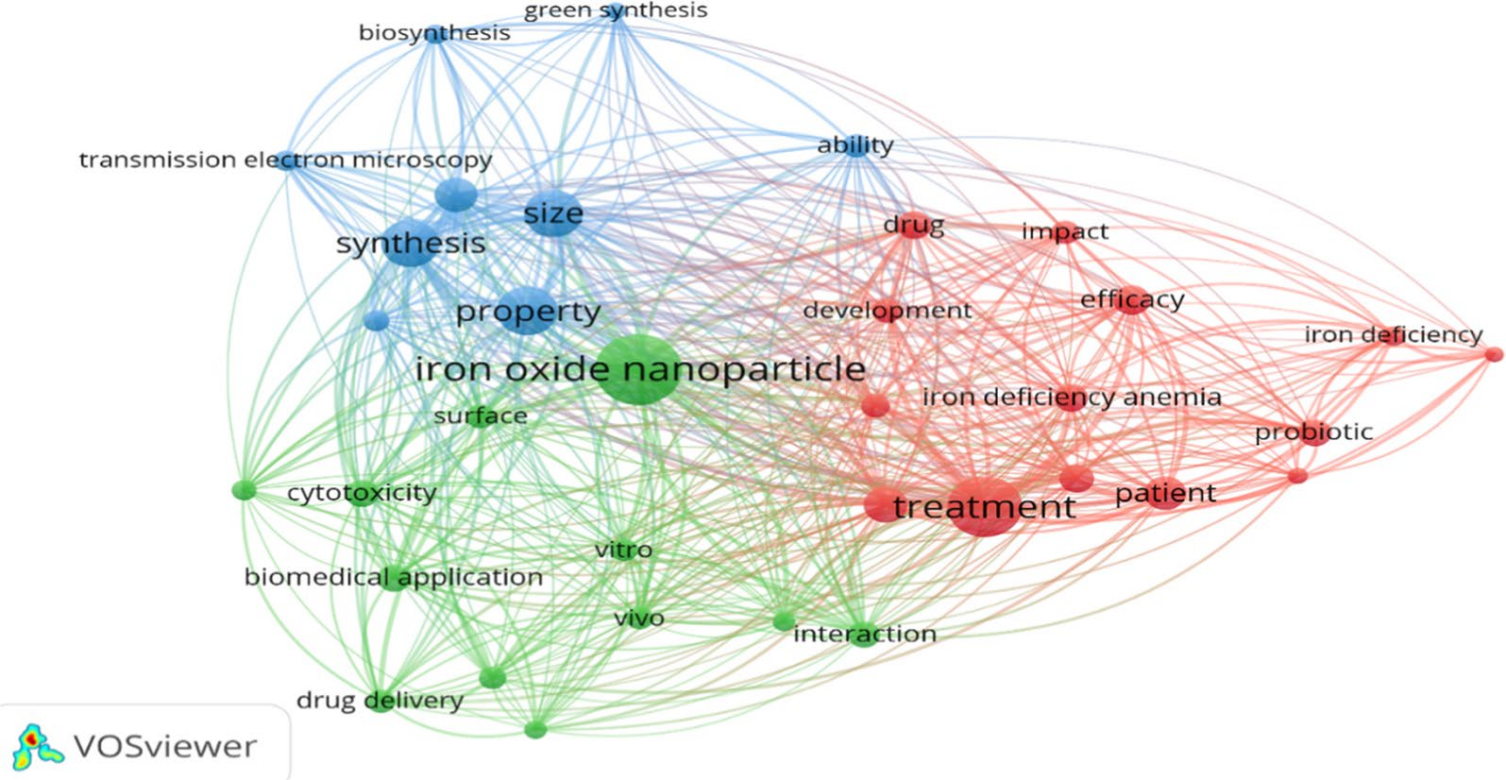
Analysis of IONP synthesis and features using anemia-related keywords (VOSviewer version 1.6.17). The connecting lines highlight the relationship between the different properties of IONPs and their effect on the treatment of IDA

In addition, the VOSviewer program was used to provide an overview of the interaction between IONPs, probiotics, and IDA by analyzing the main keywords of the included studies (Fig. 2).

### Qualitative analysis

The authors examined the risk of bias using the Office of Health Assessment and Translation (OHAT) risk of bias rating tool for human and animal studies. Based on knowledge of the current human exposure levels, the OHAT risk of bias tool is designed to assess the methodological quality, sensitivity, and validation of techniques utilized, as well as the degree of variance in subjects, including mechanistic (e.g., in vitro and in vivo) studies.

**Fig. 3:**
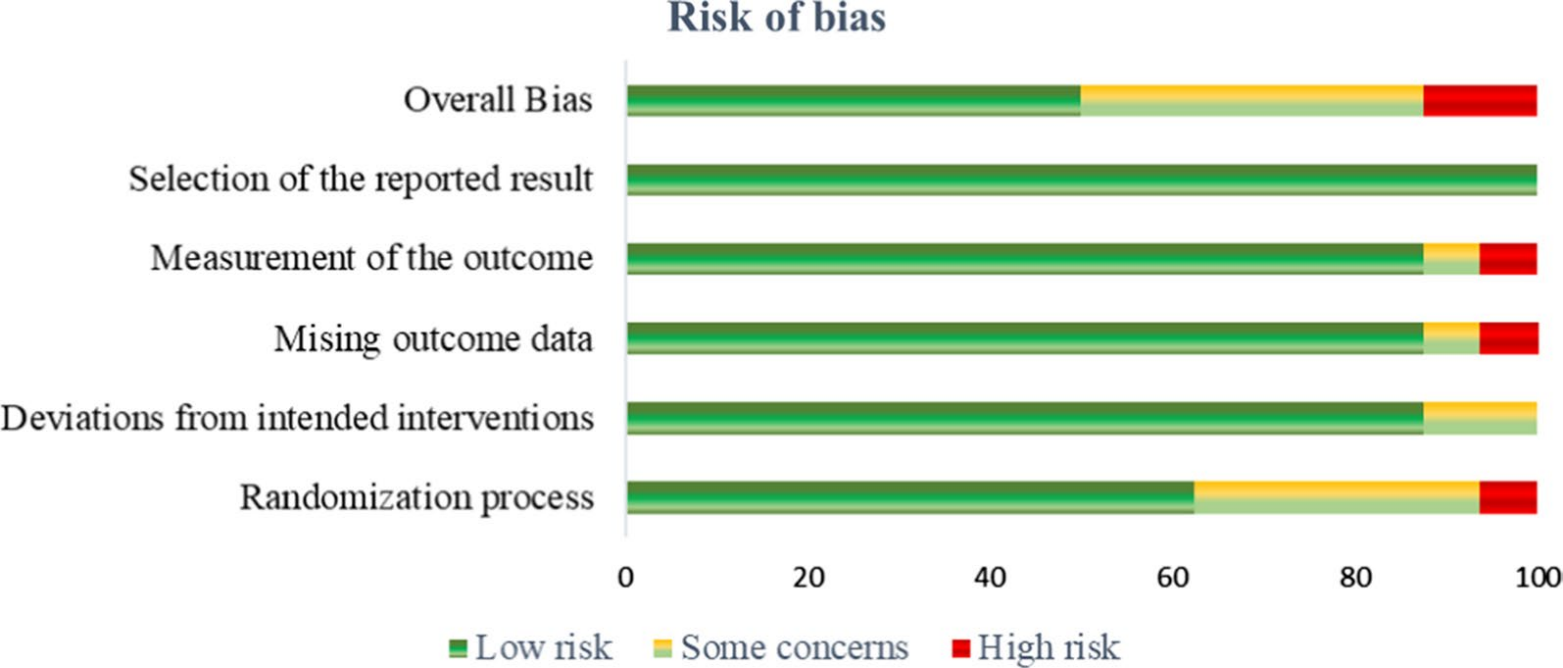
Diagram indicating the risk of bias of the included studies

The quality of evidence was based on the evaluation of the publications by their sustained conclusion, number of reported exposure conditions, and concordance across the results. Among the overall bias, 12.5%, 37.5%, and 50% of studies were classified as having a high, medium, and low risk of bias, respectively (Fig. 3).

### Quantitative analysis

#### IONP synthesis

The electronic, optical, and magnetic characteristics of IONPs confer good potential in many areas, such as biomedicine, nanobiotechnology, material science, chemistry, and physics [22, 40–42]. The beneficial effects of IONPs in vitro, in vivo, and in clinical trials have been demonstrated in 60 studies considered here for their synthesis. However, the toxicity of IONPs is mainly established from their physical and chemical characteristics, which are derived from their synthesis method [42, 43]. Various synthesis methods exist, including chemical, physical, biological (green), and hybrid strategies.

*Physical methods* Researchers have investigated the development of efficient methodologies for IONP synthesis based on their controlled shape and size, biocompatibility, and monodisperse nature [41, 44–48]. The methods drastically affect the structural and morphological characteristics of the IONPs; therefore, the magnetic and chemical surface properties significantly determine or tune their application in various multidisciplinary areas. One example of a physical synthesis method is a laser-based method that applies aerosol organometallic precursors [49]. By varying the concentration of the benzoic acid in the solution and employing pre-stabilized mannitol IONPs, nanoparticle size can be controlled [50]. Laser ablation synthesis, which occurs when a pulsed laser fascicle interacts with a target material immersed in a liquid solution; this route can produce metal nanoparticles without any chemical stabilizers, although the size and shape are difficult to control [51]. Recent experiments revealed that colloidal dispersions of IONPs were generated when phosphonates were added as an ablation medium [52, 53], with the composition and crystalline stability variations were observed as a function of the size of the nanoparticles and the laser wavelength [54]. A protective oxide coating was also designed using Fe_3_O_4_ and/or Fe_2_O_3_ [55]. This method is economical, simple, and environmentally friendly [52, 53]. IONPs may be a promising technology for producing oxide bimetallic nanoparticles because they are generated directly in a liquid medium without contamination [54]. Several characteristics were examined, including the effect of pH, H_2_O concentration, and recyclability. The 3D hierarchical nanostructures of the iron oxide coatings were shown to improve activity and mechanical stability. Stress-induced phase segregation was suggested to occur during thermal annealing as the growth process for nanostructures [53].

*Chemical methods* The chemical methods used for IONP synthesis, as detailed in Table 1, include precipitation/coprecipitation, hydrothermal, microemulsion, combustion, and sol–gel reactions [56–59]. The associated research emphasizes the effects of various reaction conditions that would lead to the generation of nanomaterials with the smallest size, a high degree of dispersion, a well-defined structure, and achieve efficient control over the characteristics.

**Table 1:** Synthesis of IONPs via (a) precipitation, (b) hydrothermal, (c) microemulsion, and (d) sol–gel methods

Chemical synthesis method	Characteristics of the synthesis	Size distribution	Shape	Ref.
Precipitation	Simple method, fast reaction, high yield Possible risks to the environment and living organisms	Reduced control	Irregular shape	[17, 43, 46, 72, 75]

Hydrothermal	Elevated temperatures in an inert atmosphere High degree of crystallinity Long reaction time	Uncoated nanoparticles; tendency toward agglomeration	Spherical shape	[40, 58, 132]

Microemulsion	Ambient temperatures for the reaction, low yield, highly uniform morphology Large quantity of solvent	Narrow size distribution	Spherical shape	[56, 60]

Sol–gel	Simple method, high yields Fast preparation, formation of safe byproducts	Narrow size distribution	Quasi-spherical shape	[57, 59]

Briefly, the salts of Fe^2+^ and Fe^3+^ ions are exposed to either a basic solution (precipitation) [43], a constant isotropic solution of oil and water (microemulsion) [60], or vapor in a sealed container (hydrothermal) [57] under specific temperature and pressure conditions. The efficacy of the precipitation method has extensively studied because of the toxicological effects and health hazards caused by nanoparticles [43]. Glycyrrhizic acid (GA)-coated IONPs, which are produced via oxidative precipitation, are suggested to be anticancer agents with low cytotoxicity and increased biocompatibility [47]. However, chemically prepared IONPs using precipitation were found to be more toxic to the kidneys and epithelial cells of Wistar rats compared with nanoparticles prepared via the green synthesis method, because of inadequate crystallinity [43]. Thermal decompositions can also be used to adjust the size of magnetic IONPs [61]. The reaction involves a pressurized system to heat the solvents above their boiling points [62]. This process requires significantly more expensive and toxic precursors and organic surfactants [46]. Hydrolysis, particle growth, condensation, and particle agglomeration are the four key steps in the sol–gel procedure, which achieves connectivity in the continuous liquid phase by colloidal suspension (sol) and gelatin (gel) [57]. This is the most straightforward method, in which constant monitoring of the reaction parameters can be used to control the particle size and shape [30]. Microemulsion methods are ideal for producing crystalline inorganic nanoparticles [60]. For example, simple synthetic conditions at (near)-ambient temperatures and pressures facilitate the synthesis of a large variety of nanomaterials, with reasonable control over size, shape, and composition. Owing to their superparamagnetic properties and biocompatibility, magnetic hybrid nanogels constituted from magnetic nanoparticles and a polymer of hydrogel matrix have attracted attention [63]. Lower critical solution temperature-driven self-assembly and the cross-linking of IONP-grafted polymers were employed to cluster the IONPs inside the fluorescent polymer nanogels [64]. However, despite its efficiency, it is difficult to scale up this approach because of the large solvent volumes required [56].

**Fig. 4:**
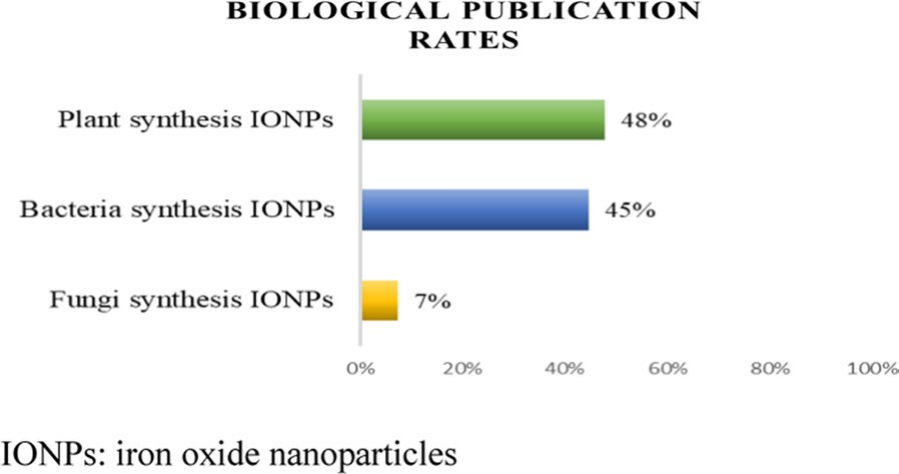
Publication rates (2017 to 2021) including the terms “fungi,” “bacteria,” and “plants” connected to IONPs. (Source: Scopus, searched on 10 January 2022). *IONPs* iron oxide nanoparticles

*Biological methods* Biological interfaces provide a promising new path for synthesizing environmentally friendly multifunctional IONPs [17, 22, 23, 43, 65–72]. Figure 4 shows the number of articles that were retrieved from the Scopus database (2017 to 2021) using the keywords “fungi,” “bacteria,” and “plants” related to IONPs in the title, keywords, or abstract. The most prevalent size-reducing intermediaries used to develop nanoparticles are plants (48%), followed by bacteria (45%) and fungi (7%). The approaches are based on the utilization of plant extracts or microbial-derived compounds with a reduced ability to connect with iron precursors [41, 42, 73, 74]. For example, the use of leaf extracts of *Ruellia tuberosa* [16], *Moringa oleifera* [66], *Sageretia thea* [41], and *Petroselinum crispum* [75] in IONP synthesis could assist in killing pathogens (*Escherichia coli* [76], *Klebsiella pneumonia* [77], and *Staphylococcus aureus* [78]) and enhance the biodegradability of industrial wastewater [16, 79]. These methods are economical if precipitation is the primary procedure [41, 43, 80].

Reports on IONP biosynthesis are shown in Table 2. IONP synthesis from hydroponically generated spinach extract yielded an iron concentration of 40.34% compared with only 0.0007% ppm in the comparable plant extract. This process yielded spherical nanoparticles with a diameter of 10–50 nm [71]. Smaller IONPs (6.22–9.7 nm) were obtained by chemical synthesis compared with the IONPs synthesized using *Petroselinum crispum* leaves extract (64–68 nm) [75]. The peel extract of *Punica granatum* fruit reduced the size of IONPs to < 11 nm and IONPs containing 2–4% peel extract had significant anticancer activity against the HONE1 nasopharyngeal carcinoma cell line [81]. Iron-reducing bacteria, such as *Aspergillus niger* [74], *Trichoderma asperellum*, *Phialemoniopsis ocularis*, *Fusarium incarnatum* [23], *Bacillus subtilis* [80], *L. casei* [67], and *L. fermentum* [33], can be used for IONP biosynthesis. Some extracellular enzymes have excellent redox properties in bacteria, thereby serving as a biological nanoreactor and acting as an electron shuttle in the reduction of metal ions to form nanoparticles and stabilizing them with a covering agent [80]. Fe_3_O_4_ nanoparticles are not stable during biosynthesis conditions; they can be rapidly oxidized to Fe_2_O_3_ or dissolved in acidic media, resulting in the control of the surface charge by the pH [45]. An *L. casei* extract was used for producing very small, spherical IONPs [67]. Synthesis methods for IONP production by bacteria are biologically safe, low-cost, simple, and environmentally friendly [33].

**Table 2:** Biosynthesis of IONPs

Reducing agents	Species extract	Synthesis parameters	Shape	Size (nm)	Matrix	Effects	Ref.
Plant	*Stevia rebaudiana Bertoni*	13 h at 170 °C	Spherical	20–25	DPPH radical	↑ Antioxidant activity	[68]
	*Punica granatum*	45 min at 25 ºC pH 11	Spherical	26.52–158.44	Cancer cell lines	↑ Purity and crystallinity of IONPs ↑ Denaturation of the HONE1 NPC cell line ↓ Cytotoxicity of CCD112 and HEK293 normal cells	[81]
	*Petroselinum crispum*	2 h at 25 ºC	Oval cubic spherical	64–68	Male albino rats	↓ Serum ferritin and iron concentrations ↑ Total iron-binding capacity, urea, and creatinine	[75]
Bacteria	*Paenibacillus polymyxa*	5 h at 45 ºC pH ± 4.8	Spherical	26.65	Maize seedling growth	↑ Seed germination, root development, and fresh weight	[70]
	*Enterobacteriaceae*	10 days at 25 ºC pH 7.4	Spherical	0.9–1.8	Hep-G2 hepatocarcinoma cell lines	↑ Cell viability after 24 h (500 μg/mL)	[2]
					Sprague Dawley rats	↑ Content of iron in serum and tissue, as well as the expression of the ferritin L subunit	
	*Pseudomonas aeruginosa*	48 h at 37 °C pH 6.5	Spherical	23	Human plasma	↑ Anticoagulant activity in the final common pathway and in the intrinsic pathway of the coagulation process (determination of APTT) ↓ Anticoagulant activity in the extrinsic pathway	[45]
Fungi	*Trichoderma asperellum*	5 min at 30 °C pH 3.2 ± 0.02	Spherical	25	Fungal cell filtrate	↑ Stability in nature ↑ Hydrolysis potentiality of iron chloride salts ↑ Extracellular nanoparticle formation	[23]
	*Phialemoniopsis ocularis*			13.13			
	*Fusarium incarnatum*			30.56			

*DPPH* 2,2-Diphenyl-1-picrylhydrazyl, *NPC* nasopharyngeal carcinoma, *APTT* activated partial prothrombin time, *IONPs* iron oxide nanoparticles

#### IONPs—Metabolism and cellular absorption

**Fig. 5:**
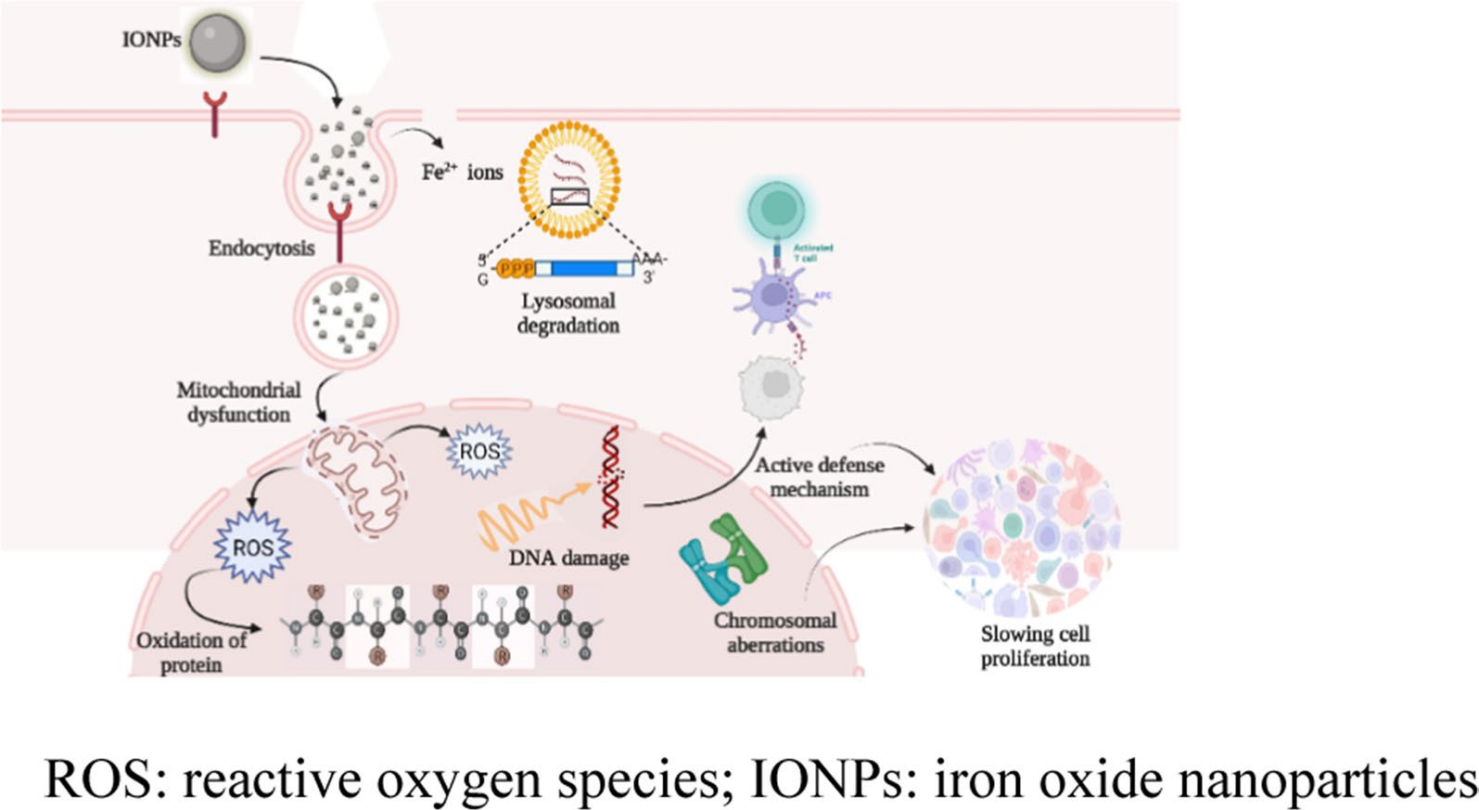
Tentative schematic describing IONP-induced toxicity on the cellular level. *ROS* reactive oxygen species, *IONPs* iron oxide nanoparticles

The term bioavailability describes to the ability of the human body to absorb a given compound [8, 82]. Iron is involved in vital biochemical activities, such as metabolism, biosynthesis, replication, transport, and enzymatic reactions involving cytochrome, dopamine, and hemoglobin [8]. Dietary iron has two forms: heme (Fe^2+^) and non-heme (Fe^3+^) [83]. The former, with high bioavailability (25–30%), comprises hemoglobin and myoglobin [8, 83]; the latter, which can be obtained from plant and animal sources, differs in chemical structure, absorption methods, and uptake mechanisms, and has low bioavailability (1%–10%). Fe^3+^ can only be absorbed if converted to ferrous iron (Fe^2+^) in the presence of the duodenal cytochrome b reductase 1 (DCYTB) [83]. Reducing agents, such as ascorbic acid, citric acid, other organic acids, and amino acids (cysteine and histidine), may increase endogenous stomach acid production, thus stimulating iron absorption [84]. Dietary nutrients such as ascorbic acid and meat improve non-heme iron absorption [85]; polyphenols, calcium, and phytic acid hinder it [8]. The duodenum and upper jejunum are significant areas for intestinal iron absorption (90%), whereas the stomach accounts for < 2% of this process [8, 86]. Duodenal enterocytes absorb the resulting iron (Fe^2+^) through the divalent metal transporter 1 (DMT1), where it may be stored as ferritin, utilized to produce iron-containing proteins, or transported to the plasma through the membrane protein ferroportin [86]. More than 25% of the body’s iron is deposited in the liver, spleen, and bone marrow as a complex with hemosiderin, ferritin, and transferrin [87]. To increase iron absorption, many researchers highlighted the use of IONPs in the management of IDA [2, 5, 75, 88, 89]. Nanoparticles can cross the plasma membrane during in vivo and in vitro cell exposure using various distinct cellular entrance pathways; these can be classified into two groups: (i) endocytosis-based absorption pathways and (ii) nanoparticle direct cellular entrance [90]. Figure 5 shows the interaction between IONPs and biological cells. IONPs can destabilize homeostasis at different levels [91].

Following oral administration, IONPs elevate iron levels in the spleen and liver in vivo, indicating that some particles pass the intestinal walls [24]. It is suggested that IONPs injected into the bloodstream are absorbed by macrophages in the organs of the mononuclear phagocyte system, resulting in their removal from the blood circulation [92]. Endocytosis is the most common process of IONP absorption and allows access to endosomal division, regardless of nanoparticle dose and exposure period [93]. This fact explains the cellular heterogeneity of nanoparticle distribution and permits the establishment of simple but strong probability distributions that correctly forecast the nanoparticle dosage to individual cells [94]. Endocytosis of nanoparticles mainly occurs via phagocytosis, clathrin-mediated endocytosis, caveolin-mediated endocytosis, independent clathrin/caveolae endocytosis, and micropinocytosis [95–97]. The strategy through which the nanoparticles enter a cell strongly relies on the cell type [98]. Initial endosomes connect with endocytic vesicles, directing nanoparticles to specific cellular areas. The clustering and binding of nanoparticle surface ligands to homologous cell membrane receptors initiate clathrin-dependent endocytosis, a major mechanism for nanoparticle cellular entry [96].

After exogenous materials enter living organisms, the immune system responds differently; neutrophils either inactivate them by degranulation, generating reactive oxygen species (ROS), or immobilize them by producing chromatin with cytoplasmic granular proteins as neutrophil extracellular traps (NETs) [95]. Biocompatible human serum albumin or dextran coatings, which are used for nanoparticle stabilization, decrease agglomeration and NET formation [95]. The nanoparticles that follow direct translocation paths may break the cell plasma membrane by interacting with the lipid bilayer molecules that transport them directly into the cytoplasm [90]. Therefore, using the cell-penetrating peptides as nanoparticle surface ligands is an alternative technique [99]. When IONPs are inserted into living organisms and encounter biological fluids, their surface immediately interacts with proteins and other macromolecules, producing a “protein corona” that can radically affect the aggregation state, nanomaterial size, and interfacial characteristics, thus influencing the uncontrollable biological behavior of IONPs [100]. Thus, this protein corona is primarily responsible for IONP disposition and is involved in slowing the nanoparticle degradation process [101]. However, significant deviations in IONPs with a corona produced from human plasma were detected as a function of the lipid adsorption profile [102]. IONPs were reported to be associated with inflammation and pulmonary oxidative stress [103]. Severe exposure of lung epithelial cells to IONPs may modify the cell biomechanical properties and potentially impairing the integrity of the epithelial barrier [28].

#### Cytotoxicity of IONPs

The unique qualities of IONPs has increased their prominence as potential catalysts in the ongoing scientific and technological revolution [71, 80, 87, 102, 104, 105]. Despite their advantages, in vivo and in vitro toxicity associated with IONPs has been reported in human cells [106, 107]. Therefore, it is critical to determine how IONP-based drug carriers are metabolized, degraded, and/or successfully eliminated after drug release at the target tissue [91]. The cytotoxicity of IONPs can be attributed to the high amount of Fe^2+^/Fe^3+^ ions exposed on the large surface area of the nanoparticles, as well as their aggregation, which impacts their distribution and removal, and may lead to excessive cellular accumulation [30, 105]. The generation of ROS is a source of cellular oxidative damage in cells (lipids, proteins, and DNA) [108]. The principal factors that can impact the toxicity of IONPs are shape, size, hydrophobicity/hydrophilicity, surface charge, core composition, and coating [30, 105, 106, 108] (Table 3). Particles smaller than 10 nm have a large surface area to volume ratio, resulting in a greater number of surface atoms that can quickly oxidize to Fe^3+^, generating Fe_2_O_3_ on the magnetic particle’s surface [92]. Biocompatible ligands, which include organic acids with a low molecular weight, natural amino acids, or tartaric/adipic acid, can be used on the surface of the nanoparticulate materials to generate biocompatible and nontoxic IONPs [29, 65]. Dextran, polyvinylpirrolidone (PVP), polyethylene glycol, and other coating materials have been utilized to modify the surface chemistry of IONPs [25, 26, 99, 107, 109]. For PVP coatings, dose-dependent cytotoxicity was detected [26, 110]. The hydrophobic surfaces of uncoated IONPs facilitate their aggregation owing to high surface-to-volume ratios [106]. In addition, magnetite-containing compounds (Fe_3_O_4_) coated with pectin and bacteria exhibited the lowest decrease in viability in saliva and gastric media, owing to the lyophilization process, which allowed the magnetite–pectin layer to cover its entire surface, preventing the activation of dioxygen in the degradation process [111]. Apoptosis was associated with a dosage- and time-dependent administration [92], which might indicate the induction of ROS formation and DNA damage [112]. In vivo, IONP administration (0.15 mg/L) to fish yielded considerable histological alterations in the liver, including sinus hyperemia, hepatocyte vacuolization, psychosis, hepatic lobule disruption, and atrophy [104]. The detrimental effects of IONPs on carps were mitigated by the addition of *L. casei* to their diet, with a significant reduction observed in severe histopathological effects [113].

**Table 3:** In vivo and in vitro toxicological analysis of IONPs

Study Model	Size (nm) shape	Synthesis approach	Coating	Concentration	Cell line/in vivo model	Toxicity	Ref.
In vitro	50–150 Rod and spherical	Biological	Uncovered	200 µg/mL	Hepatocellular carcinoma (HepaRG) and Caco-2 cells	Caco-2 cells showed no changes in ROS, apoptosis, or mitochondrial membrane potential Two types of particles activated apoptosis in HepaRG cells, and one changed the mitochondrial membrane potential at non-cytotoxic doses	[24]
	45 Rod	Chemical	Βcd	40 µg/mL	Fibroblast cell line (NIH 3T3)	Over 24 h, Prussian blue staining indicated complete uptake of IONPs βCD-IONPs had minimal toxicity in the NIH 3T3 cell line Dose-dependent cytotoxicity of bare IONPs	[110]
	20 Spherical	Chemical	PVP	1–100 μg/mL	Human neuroblastoma, SHSY5Y cell line	The mitochondrion was the first organelle affected at the cellular level in these human neuronal cells, after only 48 h The cellular membrane of SH-SY5Y cells was not degraded	[26]
	10	Chemical	Polyethyleneimine-interfering RNA	10–80 μg/mL	HSC-T6 cell lines	Very low toxicity to HSC-T6 cell proliferation was observed	[25]
	7–22 Polygonal	Biological	Oleic acid	5, 10, 25 µg/mL	Human keratinocytes HaCaT cells	Absence of toxicity to human keratinocyte viability, proliferation, and migration	[44]
	4	Chemical	Tartrate-adipate	0–4000 µmol/L	HT-29/Caco-2 cells	In vivo investigations in the small intestine revealed a 79.3% absorption rate	[29]
	5–10 Agglomerates	Chemical	Dextran	10–100 μg /mL	Human monocytes	No cytotoxicity detected Human monocyte viability was improved; however, the underlying mechanism remains unclear	[99]
	50 Globular	Biological	Natural amino acids	49–373 μg/mL	HFF2 cell line	Nontoxic and biocompatible These nanoparticles have potential uses in cellular labeling, drug and diagnostic delivery, and other biomedical applications	[65]
In vivo	6.2 ± 1.1 8.5 ± 1.6 Spherical	Chemical	Dextran Uncoated	0.1–100 μg/mL	Zebrafish (*Danio rerio*)	Uncoated IONPs at doses of 5 and 50 g/mL were very toxic to zebrafish embryos, causing death. Locomotor behavior appeared to be unaffected by uncoated IONPs Zebrafish larvae with damaged locomotor activity better absorb lower doses of dextran IONPs (1 g/mL)	[107]
	10	Chemical	SPION-PEI/siRNA	3 mg Fe/kg	Sprague Dawley Rats	SPION-PEI/siRNA complexes were particularly abundant in the liver and spleen, whereas iron was almost absent in the heart, lungs, and kidneys	[25]
	7–22 Polygonal	Biological	Oleic acid	300 µL	Hairless mice SKH-1	Acute dermal toxicity study outcomes revealed some alterations in physiological skin parameters, albeit at levels that were not sufficient to compromise the skin barrier function	[44]
	100 50 30	Chemical	Phospholipid Dextran Uncoated	6 mg/day	Piglets (males)	No signs of iron toxicity for a variety of toxicological indicators that could suggest the occurrence of oxidative stress or inflammation Promising nutritional iron supplement	[10]
	45 Rod	Chemical	β-cyclodextrin	2000 mg/kg	Wistar rats	No significant cellular toxicity was observed after 14 days of exposure	[110]
	4	Chemical	Tartrate-adipate	35.6 ± 0.6 mg/kg	Wistar rats	The duodenum plays an essential role in iron absorption, with up to 38% and 62% greater iron intake in this region than in the jejunum and ileum, respectively Low cytotoxicity and ROS generation were identified, indicating only minor increases in free radical production The bloodstream appears to play a role in the systemic biodistribution of IONPs to organs such as the spleen, liver, and kidneys	[29]

*ROS* reactive oxygen species, *IONPs* iron oxide nanoparticles, *βCD* β-cyclodextrin, *SPION-PEI/siRNA* Polyethyleneimine designed for small interfering RNAs

Histological investigations evaluated the toxic effects of biosynthesized IONPs at various doses (10–100 mg/kg) in Wistar albino rats with IDA [71]. Conversely, the administration of IONPs at 1000 mg/kg to rats for 28 days promoted hepatic portal system congestion without affecting the kidneys or the brain [27]. The cytotoxicity of metallic nanoparticles is associated with potential ion emission and oxidative damage properties [26, 96, 99, 110]. Although there is limited knowledge on the toxicological status of IONPs, many factors, such as dose, structure, and physicochemical properties, can present danger to humans and animals.

#### IONPs carried by probiotics

Because iron is the principal component of hemoglobin, myoglobin, and several enzymes, iron deficiency is connected to lower resistance to infection, reduced productivity, fatigue, and fetal mortality [89]. Currently, oral FeSO_4_, fumarate, or gluconate, in various doses and frequencies, are prescribed for the management of IDA [90, 114]. Moreover, 89.2% of women with anemia treated for 8 weeks with ferrous bis-glycinate (27 mg/tablet) had hemoglobin levels of > 11 g/dL compared with 71.3% in those treated with FeSO_4_ glycine (100 mg/capsule) [115]. Unfortunately, severe GI tract-related side effects can occur, such as constipation, diarrhea, and nausea. Iron salts also induce alterations in food color and taste [4, 115]. Conversely, chelated iron preparations, including amino acids, probiotics, and symbiotics, produce fewer GI adverse effects and result in faster absorption [115]. IONPs have afforded considerable improvements in IDA treatment [5, 116]. Because of their higher bioavailability and effectiveness in accessing tissues, IONPs have emerged as potential iron supplements [10, 34]. In the treatment of IDA, compared with FeSO_4_, IONPs led to a significant increase in erythrocyte (RBC) counts and indices, hemoglobin concentration, compact cell volume, ferritin, hematocrit (Hct), transferrin saturation, and total iron-binding capacity (TIBC) [89]. The hemoglobin, RBC, and Hct values in IDA rats treated with a dose of 2.0 mg/kg/day astragalus polysaccharide core IONPs revealed the significant therapeutic impact of these agents [5, 9, 115, 116].

*Binding mechanism* IONPs have limited potential as fortifiers owing to their limited colloidal stability and high oxidation/aggregation rates in solution [20]. This can be resolved by surface modification (bio-organic) or introducing hydrophilic groups [10, 25, 26, 89, 107]. Among IONPs, polysaccharides offer the advantages of water solubility and stability [116]. Organ toxicity is thought to be reduced when nanoparticles are encapsulated in a liposome [89]. Conversely, many researchers have investigated the use of different probiotics to ameliorate the side effects of IDA therapy [12, 117]. An in vitro study of the effect of probiotics on intestinal iron absorption showed that the molecules released by these bacteria convert Fe^3+^ to Fe^2+^, which could imitate the action of DCYTB in the digestive system [8]. After release into the environment, IONPs more effectively interact with biological matrix/fluids because of their size, leading to high reactivity and changes in the environment and fundamental structure of the nanoparticles [102]. When IONPs, which are positively charged, approach bacterial cells, they promote electrostatic interactions with the negatively charged components of the bacterial cell membrane, such as lipopolysaccharides, lipoteichoic acids, proteins, and phospholipids via the positive charge of IONPs [36]. Moreover, IONPs can stimulate or inhibit microbial growth depending on the type of bacteria and the proportion of nanoparticles [118]. Even though iron is not a growth factor for lactic acid bacteria, high dosages of IONPs tended to increase viability of *L. rhamnosus* [36, 119]. As shown by TEM images, when Fe_2_O_3_ nanoparticles with different shapes were homogenized in *S. thermophilus* and *L. acidophilus*, most of the magnetic nanoparticles become connected to the exopolysaccharides of bacteria. The presence of nanoparticles has no detrimental effect on the reproduction capacity of bacteria; thus, this combination can be incorporated into fermentative foods, for example, as an IDA treatment [118]. Probiotics can protect other organs by absorbing IONPs, which increase iron absorption in the small intestine [35, 119] and decrease the risk of IONP-related toxicity [12, 26, 36, 92].

*Ingestion* Nanoparticles may enter the body via different routes, including oral intake, inhalation, dermal or ocular penetration, and injection [25, 29, 92, 95]. Oral intake is the best known, because of its easiness, low risk of adverse effects, and good patient compliance [25, 29]. However, the acidic stomach of medium reduces drug stability, and the digestive enzymes can degrade the drug, thereby reducing its bioavailability. In simulated saliva, Fe_2_O_3_ was decreased by 35% nanoparticles/mL [120] when taken orally, whereas the IONPs pass through the GI tract, where the acidic stomach juice might cause their disintegration and release of ionic iron [24].

*Transport* Because of their small size, IONPs enable possible uptake in the liver, spleen, kidneys, and brain, causing cell damage and oxidative stress [24, 65, 105, 106]; therefore, knowledge of their biodistribution and toxicity is essential [25, 27, 28]. As only a small proportion of dietary iron is assimilated, high amounts are required, which requires the identification of useful transportation techniques [7, 82, 121]. Overcoming the stomach’s acidic environment remains difficult [10, 116]; in a simulated gastric fluid, IONPs (100–180 nm) were decreased by 72% particles/mL after 8 h [120]. The mission for ingested probiotics consists of surviving the gastric environment to reach the large intestines [8, 121]. However, there are various limitations to the use of probiotics in foods and beverages, such as their post-consumption effectiveness, which is directly related to the survival rate of the probiotics [122]. Coating probiotic cells in a suitable material can help ensure their survivability during industrial processing and GI transit [123]. For a defined alternative equilibrium, adding probiotics is especially significant and intriguing because tailored microbiome interventions have emerged as a possible therapy [124]. The probiotic *Roseburia intestinalis* has the potential to biomineralize nanoparticles, suggesting that probiotic cells may be able to produce long-term tailored magnetic nanostructures and endogenous magnetism, indicating the potential to treat Crohn’s disease [124]. Garces et al. [34] investigated small maghemite nanoparticles (10 nm) incorporated onto *L. fermentum* as novel iron supplements for treating rats with IDA; the results emphasized the significance of probiotics as potent oral carriers for IONPs. Maghemite nanoparticles can bypass the stomach’s acidic environment to reach the intestines, where they are taken up by enterocytes and re-balance blood parameters [34, 121].

*Absorption* For therapeutic effectiveness, two critical processes of IONPs must be controlled: biodistribution and biodegradation [115]. IONPs are transported via probiotics toward the intestines, and protective coatings can prevent their chemical degradation in the stomach [2, 47]. Probiotics such as *L. fermentum*, *Roseburia intestinalis*, *and Enterobacter* spp. serve as carriers with densely arranged magnetic nanoparticles on their exterior surfaces [34, 118, 119, 124]. Some studies suggest that the green synthesis of IONPs by probiotics has a positive effect on iron absorption [69]. The biological and physicochemical features of a nanostructured iron–polysaccharide complex (nano-IPC) biosynthesized by *Enterobacter sp*. as a supplement to counter IDA confirmed that the iron content in animal serum and tissue and the expression of the ferritin L subunit were significantly higher than following FeSO_4_ supplementation; in turn, its biochemical components and ferritin H subunit levels remained constant, indicating its nontoxic effects [2]. Increased serum and tissue iron levels are vital in erythrocytosis to achieve effective IDA treatment [69]. After 4 weeks of feeding with yogurt fortified with IONPs (*S. thermophilus*, 7.09 log_10_ CFU/g; *L. bulgaricus*, 6.88 log_10_ CFU/g; *L. acidophilus*, 6.98 log_10_ CFU/g; and *B. bifidum*, 6.74 log_10_ CFU/g), the levels of iron, ferritin, hemoglobin, and total protein were restored, although considerable competition with calcium and zinc absorption was observed [9]. Supplementation with IONPs yielded a modest increase in iron alongside by no modification in hemoglobin concentration (*P* > 0.05), whereas the intake of IONPs–bacteria restored plasma iron and hemoglobin values, similar to FeSO_4_ [34]. Interestingly, *L*. *fermentum* secreted compounds (including ferrireductase) that enable DCYTB activity, similar to the impact of administering IONPs–bacteria [8, 125]. To detect and examine the degradation of IONPs in biological tissues, the in-phase and out-of-phase temperature dependences of magnetic susceptibilities were investigated [34, 125]. Qualitatively, the IONP biodistribution appeared to be similar for ingested IONPs and IONPs–bacteria at first; however, further examination revealed greater accumulation of IONPs in the stomach and higher levels of IONPs–bacteria in the intestines, especially in the cecum, where IONPs may have decomposed faster or accumulated in a smaller proportion [125]. Because of the capacity of probiotics to interact with the intestinal walls, IONPs–bacteria are incorporated into enterocytes, where nanoparticles are delivered, thus providing adequate iron content [4, 34, 36, 118].

*Distribution* The different sizes and shapes of nanoparticles can be a factor in making the translocation from the absorption site to the circulatory and lymphatic systems, body tissues, and organs [26]. To assess the translocation process, various tissue samples were obtained at 48 h after intravenous administration of IONPs; uncoated and coated IONPs with a negative surface potential accumulated most significantly in the liver and the spleen. In contrast, the positively charged coated IONPs exhibited the highest accumulation in the lungs, indicating an accumulation in the kidneys and the blood [126]. Although the total iron in the liver did not change significantly compared with the control, TEM data confirmed the presence of the particles in the kidneys and the liver [29]. Similarly, IONPs associated with probiotics exhibited the highest deposition in the liver, lungs, and spleen, without any damaging effects or structural changes, as shown by biochemical and histological analyses [9].

*Elimination* IONP clearance requires at least 2 weeks to 6 months [92, 127]. In general, the reticuloendothelial system clears out IONPs of < 50 nm; blackfish required 15 days to remove 50% of the sequestered iron from IONPs [104]. Furthermore, evidence of IONP redistribution was obtained in time- and dose-dependent excretions in both urine and feces [128]. The clearance of feces and urine of rats was evaluated over a 5-month-period following after IONP injection. At first, the clearance profile in urine showed maximal excretion on the day after dug delivery, and was sustained until day 28, after which it declined gradually [127, 128]. Nevertheless, the iron concentration in feces remained high over the first 3 days [128], with no significant decrease up to 3 months post-injection [128].

### Limitations, controversies, and challenges

The emerging topics were critically evaluated to identify gaps in the literature regarding the medical applications of IONPs. Potential areas of study, which may be of interest to future researchers to fill in these gaps, are presented in Table 4.

**Table 4:** Gaps in the literature review

Subject	Gaps	Potential research questions
Synthesis method	In chemical/physical syntheses, surfactants, templates, and other compounds are used to stabilize and regulate the size and shape of nanoparticles with toxic potentials	What is the environmental impact of large-scale IONP production?
	Green synthesis for physicochemical and microbial stability is underexplored	Other than the implication of obtaining green synthesis through natural agents, what are the potential risks of green synthesis?
	Large-scale and reproducible synthesis	How valuable and practical are the actual synthesis methods for large-scale production?
Cellular absorption and metabolism	New ways to control nano-bio interactions in subcellular compartments	What is the minimum level of complexity for a targeted delivery system?
	Active targeting strategies	
Cytotoxicity	Limited studies have discussed the toxicokinetics and pharmacokinetics of IONPs in blood and tissues	What is the impact of IONPs on genes? How do probiotics or their metabolites impact IONP cytotoxicity?
	Few strategies are addressed concerning the tissues in which many IONPs accumulate, including the lungs, liver, spleen, and kidneys	Exactly how much of the IONPs accumulate, and in which organs?
IONPs carried by probiotics via absorption	The control over the size and shape of IONPs carried by probiotics is limited	Should researchers be worried about the safety of nanocarriers?
	Limited studies have examined the effectiveness of the addition of probiotics in nanotechnology	What are the operational and functional challenges associated with incorporating probiotics into nanotechnology?
	The bioavailability, efficacy, and adverse effects of different categories of nanoparticles with probiotics on human exposure remain unclear	How significant is the connection between the microbiome and nanoparticle applications in drug delivery?
	Evaluation of the efficacy of a method for bacterial quantification	How many IONPs may be adsorbed onto the surface of a bacterium?

*IONPs* iron oxide nanoparticles

## Conclusions

We performed an analytical and exhaustive review of the interactions of IONPs with probiotics for increased bioavailability and minimal side effects in the treatment of IDA. The required components of a systematic review consist of literature screening, search strategy, classification, and the thorough and transparent recording of all stages of the process. The inventory contained elements that considered necessary to obtain relevant information in a systematic review. The flow diagram suggested by the PRISMA standards was edited to display the number of included identified records, eliminated publications, and included studies [129].

We performed a systematic literature review on the effects of IONPs and their interaction with probiotics on iron absorption, bioavailability, microbiota balance, and associated side effects. Despite the substantial body of literature studying IONPs, the qualitative analysis of the included studies revealed the presence of substantial heterogeneity with respect to nanoparticle absorption, cytotoxicity, interaction with probiotic bacteria, storage conditions, and sample manipulation. The correlation between the nanoparticle synthesis strategy and their targeted morphological characteristics was also considered. The present work provides valuable theoretical and practical insights regarding IONPs, which were classified into four main topics. Based on the open-systems concept, we designed a framework for understanding the connection between probiotics and IONPs. This research not only summarizes the current state of knowledge, but also highlights the gaps and suggests potential novel approaches.

To the best of our knowledge, this is the first systematic study of the role of probiotics–IONPs in the treatment of IDA, which is a major health issue. Dietary iron supplementation is challenging because the conventional fortificants (FeSO_4_ and FeCl_3_) alter the organoleptic qualities of foods and induce GI distress, black stools, and other issues [130]. Barrier coatings applied to magnetic nanoparticles prevent chemical damage in the stomach, and using probiotics as transporters for intestinal delivery are options for increasing iron absorption and treat IDA [131]; however, this area of research requires further improvement. IONP-based diagnostics, medicines, and devices are expected to become common in clinical practice within the next two decades.

## Methods

### Data sources and searches

The literature search was conducted using the Boolean strategy for Web of Science, Scopus, and PubMed databases with the following keywords: nanoparticles, iron, oxide, probiotics, and absorption. This review, including reports between January 2017 and June 2022, was conducted as Preferred Reporting Items for Systematic Reviews and Meta-analyses (PRISMA) guidelines. The PRISMA statement includes 27-item criteria and a 4-section flow diagram. The inventory contained elements considered necessary to obtain relevant information in a systematic review. The flow diagram suggested by the PRISMA standards was changed to display the included number of identified records, eliminated publications, and included studies [129]. Articles written in English were exclusively considered. Systematic reviews are designed to be transparent and updatable, as well as to answer specific questions. The main question was: Can iron oxide nanoparticles transported by probiotics significantly improve iron absorption in an organism with minimum side effects? Two authors independently screened titles at first, then the abstracts. In cases of doubt, the full text was examined to confirm suitability. For eligibility, search terms and inclusion/exclusion criteria were used to select more relevant studies.

*Inclusion criteria* (1) Studies evaluating the synthesis characteristics of IONP properties; (2) in vitro*/*in vivo studies investigating the effects of IONP-delivering drugs (efficacy and/or safety); and (3) articles with reports on the targeting and absorption of IONPs carried by probiotics.

*Exclusion criteria* (1) Studies without a control group to evaluate the effect of IONPs on the absorption rate; (2) studies that focused on the correlation between IONPs and other bacteria without a probiotic effect; (3) studies that focused on the probiotic effects of another nanoparticle; (4) duplicated research articles with identical authors, title, issue number, volume, and digital object identifier; and (5) thesis papers, conference reports, editorials, and theoretical publications.

*Quality assessment* Finally, authors examined the risk of bias with the OHAT (Office of Health Assessment and Translation) Risk of Bias Rating Tool for Human and Animal Studies. To determine if these materials may be of concern, given what is known about current human exposure levels, the OHAT risk of bias tool was designed to assess methodological quality, sensitivity, validation of techniques utilized, and degree of variance in subjects, including mechanistic (in vitro and in vivo) studies. The following categories are assigned:

 “Definitely low risk of bias,” direct indication of low risk of bias practices.

 “Probably low/high risk of bias,” circumstantial/indirect evidence of increased risk of bias practices.

 “Definitely high risk of bias,” direct evidence of high risk of bias practices.
